# Discordant structural chromosomal aberrations in chorionic villi and amniotic fluid leading to a formation of an isochromosome 21: a case report

**DOI:** 10.1186/s13039-021-00549-y

**Published:** 2021-06-14

**Authors:** Eini Westenius, Maria Pettersson, Erik Björck

**Affiliations:** 1 Department of Molecular Medicine and Surgery, Karolinska Institutet, Stockholm, Sweden; 2 Department of Clinical Genetics, L4:03, Karolinska University Laboratory, Karolinska University Hospital, 171 76 Stockholm, Sweden

**Keywords:** Abnormal karyotype, Amniocentesis, Chorionic villi sampling, Chromosome aberrations, Confined placental mosaicism, Isochromosomes, Mosaicism, Prenatal diagnosis

## Abstract

**Background:**

Fetoplacental discrepancies occur in approximately 1–2% of analyzed prenatal cases. They are typically due to confined placental mosaicism, where an aberration is observed in the placental cells but not found in the fetal cells. Confined placental mosaicism usually involves aneuploidies and more sparsely structural chromosomal aberrations. To the best of our knowledge, this is the first reported case of a discrepancy in the analyses of chorionic villus sampling and amniocentesis involving two different structural chromosomal aberrations of chromosome 21.

**Case presentation:**

We report a 33-year-old woman who was referred for a non-invasive prenatal testing due to an increased risk of trisomy 21 gleaned from a combined ultrasound and blood test. The non-invasive prenatal testing showed an increased risk of trisomy 21 with a normalized coverage signal that did not match the fetal cell-free DNA fraction. Rapid aneuploidy detection performed on uncultured chorionic villi indicated mosaicism for trisomy 21. The follow-up analyses revealed discordant chromosomal aberrations: 46,XY,der(21)t(10;21)(p11.21;q10) in the analysis of the chorionic villus sampling and 46,XY, + 21,der(21;21)(q10;q10) in the analysis of the amniocentesis. Thus, the analyses indicated mosaicism for a cell line containing trisomy 21 and a cell line containing a partially duplicated short arm of chromosome 10 in the chorionic villi and complete trisomy 21 resulting from an isochromosome 21 in the amniotic fluid. The analyses of the lymphocytes and the fibroblasts of the woman were normal.

**Conclusions:**

We propose a multiple-step mechanism as a possible theoretical explanation for the formation of these discordant structural chromosomal aberrations in the chorionic villi and amniotic fluid. With this case report, we want to highlight the importance of understanding the possible underlying embryological mechanisms when interpreting results from different prenatal analyses.

## Background

Trisomy 21 (Down syndrome) is the most common chromosomal abnormality at birth and in 2019 was detected in 1:323 pregnancies in Sweden [1]. About 90–95% of all trisomy 21 cases are due to the presence of an additional free chromosome 21 in all cells, 2–4% are due to mosaicism, and approximately 2–4% are caused by translocations involving chromosome 21 [2, 3, 4, 5]. The most common acrocentric rearrangements involved in trisomy 21 are Robertsonian translocations between chromosome 14 and 21, and between both chromosomes 21 [3, 6, 7]. A proportion of the latter are heterodisomic and thus represent real Robertsonian translocations with two long arms from different chromosome 21, while the majority are isodisomic with two identical long arms of chromosome 21 constituting an isochromosome 21 (i(21q)) [8, 9, 10]. Different mechanisms have been proposed for the formation of isochromosomes, including a mis-division of the centromere and a U-type exchange between sister chromatids [10, 11].

Chromosomal mosaicism develops as a result of a postzygotic mutational event [12, 13, 14, 15, 16]. The distribution of the mosaicism between the fetal and placental cells depends on the timing of the mutational event [12, 13, 14, 15, 16]. The fetus and the mesenchymal core of the chorionic villi are formed from the inner cell mass precursors of the blastocyst, whereas the cytotrophoblast layer of the chorionic villi is formed from the outer layer of the blastocyst [12]. When the mutational event occurs in the first zygote divisions, i.e. before the differentiation of the trophoblast and the inner cell mass, mosaicism can be generalized both to the placental and fetal cell lines [12, 13, 14, 15, 16]. When the error occurs at a later embryological stage, i.e. after the separation of the fetal and placental compartments, the abnormal cells may be confined to the placenta (confined placental mosaicism, CPM) or the fetus, but not necessarily both [12, 13, 14, 15, 16]. There are three different types of CPM dependent on the placental cell of origin: cytotrophoblasts (CPM type I), mesenchymal core (CPM type II) or both (CPM type III) [13, 14, 15, 16]. CPM type I can be detected through short-term culture villi, while CPM type II can only be detected after long-term culture villi [12, 13]. In CPM type III the aberration is present both after short-term and long-term culture villi [12, 13].

Fetoplacental discrepancies occur in approximately 1–2% of analyzed prenatal cases and most of them are due to CPM [17, 18, 19, 20, 21, 22]. CPM usually involves aneuploidies with autosomal trisomy being the most common, whereas structural chromosomal aberrations in mosaic form are rare and can thus represent a diagnostic challenge [12, 18, 19, 21, 22, 23].

Here we report a discrepancy in the analyses of chorionic villus sampling (CVS) and amniocentesis (AC) with two different structural chromosomal aberrations. The analysis of the CVS showed mosaicism for a cell line containing trisomy 21 and a cell line containing a partially duplicated short arm of chromosome 10, while the analysis of the AC showed complete trisomy 21 resulting from an isochromosome 21.

## Case presentation

The proband is a 33-year-old previously healthy female, gravida 1, para 0, who was referred for a non-invasive prenatal testing (NIPT) in gestational week 12 + 2 after an increased risk score was given from a combined ultrasound and blood test (risk 1:181 of trisomy 21). The NIPT showed an increased risk of trisomy 21 with a normalized chromosome value of 5.97 (normal < 4) and an increased chromosome 21 coverage of 2%. The fetal cell-free DNA fraction was 8% and thus, the expected increased coverage should have been around 4% if a chromosome aneuploidy had been present in 100% of the cells of the fetus. Hence, an aneuploidy restricted to the placenta (CPM) or fetal mosaicism was initially suspected.

**Fig. 1:**
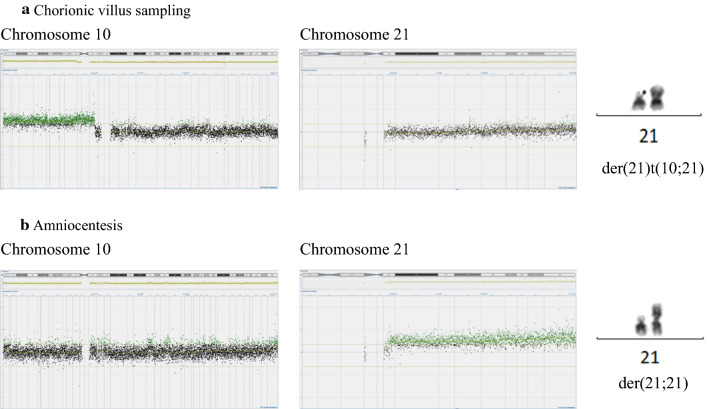
**a** Chorionic villus sampling: Array comparative genomic hybridization showed a 36.4 Mb duplication of the distal short arm of chromosome 10 (10p11.21pter) (left) and a normal chromosome 21 (middle). Chromosome analysis showed a derivate chromosome 21, der(21)t(10;21)(p.11.21;q10) (right). **b** Amniocentesis: Array comparative genomic hybridization showed a normal chromosome 10 (left) and a duplication of chromosome 21 (middle). Chromosome analysis showed a derivate chromosome 21, der(21;21)(q10;q10) (right)

As per routine, the test result was followed up with an invasive test, CVS, during gestational week 14 + 2. Rapid aneuploidy detection using quantitative fluorescent polymerase chain reaction (QF-PCR) was performed on genomic DNA extracted from uncultured chorionic villi and gave a suspicion of mosaic trisomy 21 (Table 1). The results for chromosome 13, 18 and sex chromosomes (XY) were normal. To further investigate the plausible mosaic trisomy 21 a chromosome analysis was performed. However, the chromosome analysis did not show trisomy 21, but rather an aberrant chromosome 21 that initially was suspected to be an isochromosome 21 in all cells examined (n = 26). Closer characterization of the chromosomes with fluorescence in situ hybridization (FISH) ruled out an isochromosome 21. Array comparative genomic hybridization (aCGH) performed on genomic DNA extracted from cultured chorionic villi revealed that the chromosome material attached to the chromosome 21 originated from chromosome 10p (10p11.21pter, ~ 36.4 Mb). Hence, the karyotype was established as 46,XY,der(21)t(10;21)(p11.21;q10) with a normal chromosome 21 and a duplication of almost the entire short arm of chromosome 10 (Fig. 1a). A new sample from amniotic fluid was recommended to the referring physician.

**Table 1:** Markers from QF-PCR showing the suspected trisomy 21 in mosaic form in the analysis of the chorionic villus sampling and the maternal origin of the aberrant chromosome 21

Marker	Chorionic villus sampling	Amniocentesis	Maternal blood
D21S1435 (ratio)	185/189 (1:1.3)	185/189 (1:1.8)	185/189
D21S11 (ratio)	250/252 (1.5:1)	250/252 (1.9:1)	250/260
D21S1270 (ratio)	311 & 313/319 & 322 (1:1.1)	311 & 313/319 & 322 (1:1.7)	299 & 301/319 & 322
D21S1411 (ratio)	317/330 (1.5:1)	317/330 (2.2:1)	317/322

Seven additional markers (D21S1437, D21S1409, D21S1442, D21S1280, D21S1444, D21S1246 and D21S1446) of chromosome 21, which were performed on the chorionic villi, demonstrated ratios between 1:1.3 and 1:1.5

QF-PCR and aCGH were performed on genomic DNA extracted from uncultured amniocytes. The QF-PCR showed results consistent with complete trisomy 21 (Table 1). This was confirmed by the aCGH, which detected an increased signal from chromosome 21 with trisomy 21 in 100% of the cells, but a normal signal from chromosome 10. A chromosome analysis of the amniocytes showed one normal chromosome 21 and one isochromosome 21 consisting of the long arms of chromosome 21 in all cells (Fig. 1b).

Lymphocytes and genomic DNA extracted from the peripheral blood of the woman were used for a chromosome analysis, QF-PCR, FISH and aCGH. All these analyses showed normal results. A total of 26 metaphase cells were examined for the chromosome analysis, and a total of 100 interphase cells and 25 metaphase cells were examined for the FISH analysis. Finally, in order to rule out tissue specific mosaicism, fibroblasts and genomic DNA extracted from a skin biopsy of the woman were used for a chromosome analysis (51 metaphase cells) and aCGH. These analyses also showed normal results. Markers from the QF-PCR of the woman were compared to the markers from the analyses of the CVS and the AC, and the aberrant chromosome 21 could be determined to be an isochromosome of maternal origin (Table 1). The anonymous sperm donor had a normal chromosome analysis. The pregnancy was terminated in gestational week 16 + . Gonadal mosaicism could not be ruled out and hence, the woman was counselled regarding the possibility of an invasive prenatal testing in subsequent pregnancies.

The woman became pregnant by another sperm donor approximately one year later. NIPT was performed as a first analysis and showed normal results for chromosomes 13, 18, 21 and sex chromosomes (XY). AC was recommended as a follow-up analysis and performed during gestational week 15 + 2. QF-PCR on genomic DNA extracted from uncultured amniocytes showed normal results for chromosomes 13, 18, 21 and sex chromosomes (XY). A chromosome analysis of the amniocytes showed a normal karyotype 46,XY.

A summary of the results of the different tissues in the first pregnancy is presented in Table 2.

**Table 2:** Summary of the results of the different tissues

Fetal samples	Chorionic villus sampling		Amniocentesis	
	Uncultured cells	Cultured cells	Uncultured cells	Cultured cells
QF-PCR	Mosaic trisomy 21	-	Trisomy 21	–
aCGH	–	Duplication of 10p11.21pter	Duplication of 21	–
Karyotype	–	46,XY,der(21)t(10;21)(p11.21;q10)	–	46,XY, + 21,der(21;21)(q10;q10)

Maternal samples	Blood	Skin
NIPT	Increased risk for trisomy 21	–
QF-PCR	Normal	–
aCGH	Normal	Normal
Karyotype	46,XX	46,XX

## Discussion and conclusions

We report a rare case of discrepancy in the analyses of CVS and AC involving two different structural aberrations of chromosome 21. Most of the reports regarding discrepant results in the analyses of CVS and AC are due to CPM where an aberration is only found in the placental cells but not in the amniocytes. In our report the analysis of the CVS showed mosaicism for two different aberrant cell lines; a cell line containing trisomy 21 detected by the QF-PCR and a cell line containing a partially duplicated 10p detected by the aCGH and the chromosome analysis. Meanwhile the analysis of the AC showed complete trisomy 21 resulting from an isochromosome 21.

Reports of disparities in the analyses of CVS and AC involving two or more different structural aberrations of the same chromosome are scarcely seen in literature. To the best of our knowledge, only two reports resembling ours have previously been published (19, 24). Soler et al. reported three cell lines with different structural abnormalities involving chromosome 8 [24]. In their report, CVS was performed due to advanced maternal age and a semidirect cytogenetic analysis of the chorionic villi showed mosaicism for a deletion of the short arm of chromosome 8 and an isochromosome of the long arms of chromosome 8, mos46,XX,i(8q)/46,XX,del(8)(p11.2). In the amniotic fluid, a partial duplication of the short arm of chromosome 8 was present, 46,XX,dup(8)(p23p11.2). Wang et al. in turn reported a case with discrepancies involving chromosome 18 [19]. In their report, a chromosome analysis performed on a direct preparation of the chorionic villi showed a telomeric chromosome 18 short arm, del(18)(q11), and a chromosome analysis performed on the cultured chorionic villi showed mosaicism for an isochromosome 18, either i(18p) or i(18q). The analysis of the AC showed a telomeric chromosome 18 long arm, del(18)(p11).

The underlying mechanism required to produce cell lines carrying different structural chromosomal aberrations must be complex, and is likely to involve multiple steps, as Soler et al. speculated [24]. The multistep-model Soler et. al proposed to explain their findings includes three different subsequent errors. Firstly, a meiotic error created a dicentric isochromosome 8. Next, a mis-division of the isochromosome 8 created a cell line with a deletion of the short arm of chromosome 8 confined to the trophoblast and another cell line with a duplication of the short arm of chromosome 8 confined to the fetal tissue. Lastly, an error in the trophoblast created a cell line with an isochromosome of the long arms of chromosome 8. Wang et al. in turn explained the discrepancies in their report with a transverse break of the centromere of chromosome 18 [19]. This resulted in two different cell lines: the telomeric chromosome 18 short arm and the telomeric chromosome 18 long arm. In addition, they interpreted that the presence of an isochromosome 18 in some of the chorionic stroma cells was a result of a mis-division where the two chromatids of the telomeric chromosomes replicated but failed to separate. They hypothesized that the different chromosomal aberrations migrated randomly to different fetoplacental tissues.

**Fig. 2:**
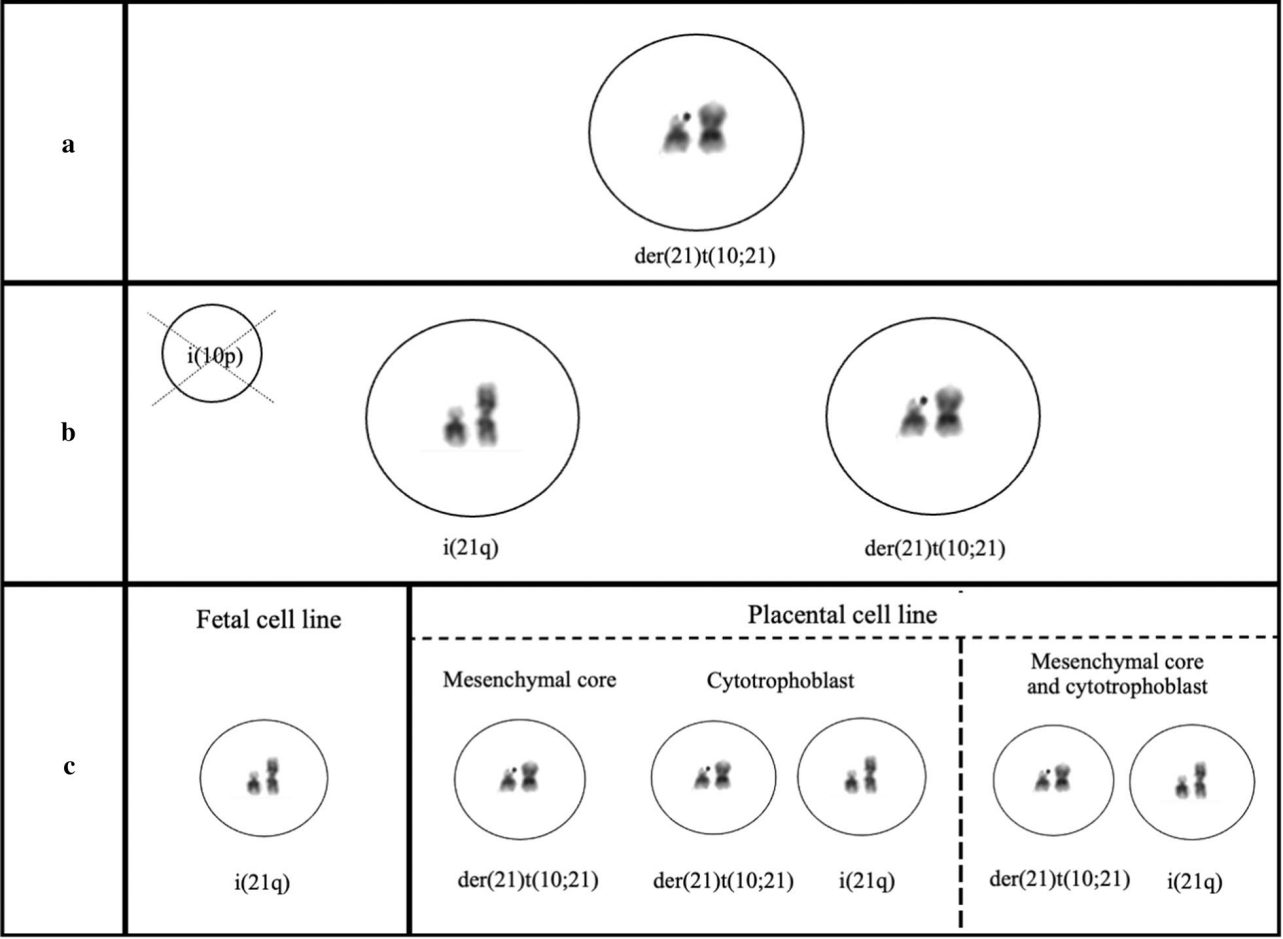
Proposed multi-step mechanism for the formation of the discordant structural chromosomal aberrations in the chorionic villi and amniotic fluid. **a** Firstly, the derivate chromosome, der(21)t(10;21)(p11.21;q10), occurred during early zygote divisions. **b** In order to rescue the chromosomal imbalance, the der(21)t(10;21)(p11.21;q10) participated in additional rearrangements in some of the cells. This led to the formation of an isochromosome 21 containing two long arms of chromosome 21, i(21q), and an isochromosome 10 containing two short arms of chromosome 10, i(10p), that was subsequently lost. **c** Lastly, the remaining chromosome aberrations were distributed into the different fetoplacental cell lines

This type of mechanism involving multiple subsequent errors in the early stage of embryonal development could offer a theoretical explanation for the formation of the different structural chromosomal aberrations in our report. It can be assumed that the derivate chromosome, der(21)t(10;21)(p11.21;q10), was the first aberration that occurred during the first zygote divisions (Fig. 2a). When this initially unbalanced rearrangement could not assume a normal chromosome structure, additional rearrangements occurred in order to try to restore the chromosomal balance. Thus, in some of the cells, the der(21)t(10;21)(p11.21;q10) was predisposed to participate in multiple additional rearrangements leading to the formation of an isochromosome 10 containing two short arms of chromosome 10, i(10p), and an isochromosome 21 containing two long arms of chromosome 21, i(21q) (Fig. 2b). The isochromosome 10 was subsequently lost. Hence, this led to mosaicism for a cell line consisting of the isochromosome 21 and a cell line consisting of the der(21)t(10;21)(p11.21;q10).

Furthermore, different cell migrations during early embryogenesis could explain the findings we reported in the different prenatal analyses (Fig. 2c). We were able to detect complete trisomy 21 resulting from the isochromosome 21 in the amniotic fluid. This indicates that the fetal cell line went through a selection process which ended in favoring the potentially more viable cell line with the isochromosome 21 and the loss of the cell line with the der(21)t(10;21)(p11.21;q10) (Fig. 2c left). There are two alternatives that could explain the formation of the placental cell lines. On the one hand, the mesenchymal core of the chorionic villi could have gone through a contrary type of selection which ended in favoring the cell line with the der(21)t(10;21)(p11.21;q10), whereas the cytotrophoblast of the chorionic villi kept both of the cell lines (Fig. 2c middle). On the other hand, all the placental cells could have kept the mosaicism for the two different cell lines (Fig. 2c right). Since the cytotrophoblast of the chorionic villi is the primary source of the fetal part of the cell-free DNA obtained from the maternal plasma, we were able to detect the mosaic trisomy 21 in the cytotrophoblasts by the NIPT. The NIPT and QF-PCR used at Karolinska University Hospital cannot detect a duplicated 10p and hence they only detected the mosaic trisomy 21 in the analysis of the CVS. As previously explained, the QF-PCR was performed on DNA extracted from the uncultured chorionic villi whereas the aCGH and the chromosome analysis were performed on the cultured chorionic villi. If the mesenchymal core of the chorionic villi went through a selection process which ended in favoring the cell line with the der(21)t(10;21)(p11.21;q10), then cell-lineage mosaicism, where the uncultured and cultured chorionic villi are primarily composed of the cytotrophoblasts and the mesenchymal cells respectively, could explain the discrepant results in the analysis of the CVS. If both the mesenchymal core of the chorionic villi and the cytotrophoblast of the chorionic villi kept both cell lines, a culture induced selection could in turn offer an explanation for the discrepancy in the results of the CVS. A culture induced selection could have led to a preferential growth of the der(21)t(10;21)(p11.21;q10) cell line detected by the aCGH and the chromosome analysis.

Another possible explanation for the formation of the chromosomal discrepancies described in our report is a vanishing co-twin, as suggested by Tharabel et al. [25]. They described a report where non-mosaic trisomy 16 was detected in the preparations of the cytotrophoblast and the mesenchymal core of the chorionic villi, whereas the analyses of the amniotic fluid and cord blood showed 46,XX. They proposed that the cells with trisomy 16 arose from residual villi that belonged to a trisomic co-twin that never developed. This mechanism is less likely, but cannot be excluded in our report either.

The limited number of reports with this type of discrepancy in the analyses of CVS and AC narrows the possibility of drawing generalized conclusions. Nevertheless, the awareness of the possibility of discrepant results in prenatal diagnostics due to underlying biological mechanisms and the limitations of different analyses is of great clinical importance, as demonstrated in our report. To better understand this type of plausible discrepancy, prenatal diagnostics must be sufficiently comprehensive, i.e. various analyses should be considered. In summary, we report, to the best of our knowledge, the first case of two different structural aberrations of chromosome 21 in the analyses of CVS and AC.

## Data Availability

All relevant data generated or analyzed during this study are included in this published article.

## Abbreviations

CPM: Confined placental mosaicism
CVS: Chorionic villus sampling
AC: Amniocentesis
NIPT: Non-invasive prenatal testing
QF-PCR: Quantitative fluorescent polymerase chain reaction
FISH: Fluorescence in situ hybridization
aCGH: Array comparative genomic hybridization
